# Mismatch Repair/Microsatellite Instability Testing Practices among US Physicians Treating Patients with Advanced/Metastatic Colorectal Cancer

**DOI:** 10.3390/jcm8040558

**Published:** 2019-04-24

**Authors:** Jennifer Eriksson, Mayur Amonkar, Gemma Al-Jassar, Jeremy Lambert, Mia Malmenäs, Monica Chase, Lucy Sun, Linda Kollmar, Michelle Vichnin

**Affiliations:** 1 ICON plc, 111 64 Stockholm, Sweden; mia.malmenas@iconplc.com; 2 Merck & Co., Inc., North Wales, PA 19454, USA; mayur.amonkar@merck.com (M.A.); monica.reed@merck.com (M.C.); linda.kollmar@merck.com (L.K.); michelle.vichnin@merck.com (M.V.); 3 ICON plc, London W12 0BZ, UK; gemma.thomson@iconplc.com; 4 ICON plc, 690 03 Lyon, France; jeremy.lambert@iconplc.com; 5 ICON plc, Boston, MA 02110, USA; lucy.sun@iconplc.com

**Keywords:** colorectal cancer, survey, mismatch repair/microsatellite instability testing

## Abstract

The study objective was to assess US physicians’ Mismatch Repair/Microsatellite Instability (MMR/MSI) testing practices for metastatic colorectal cancer (mCRC) patients. A non-interventional, cross-sectional online survey was conducted among 151 physicians (91 oncologists, 15 surgeons and 45 pathologists) treating mCRC patients in the US. Eligible physicians were US-based with at least 5 years of experience treating CRC patients, had at least one mCRC patient in their routine care in the past 6 months, and had ordered at least one MMR/MSI test for CRC in the past 6 months. Descriptive and logistic regression analyses were performed. Awareness of specific MMR/MSI testing guidelines was high (*n* = 127, 84.1%). Of those, 93.7% (119/127) physicians had awareness of specific published guidelines with majority 67.2% (80/119) being aware of National Comprehensive Cancer Network (NCCN) guidelines. Universal testing for all CRC patients was performed by 68.9% (104/151) physicians, while 29.8% (45/151) selectively order the test for some CRC patients. Key barriers for testing included insufficient tissue sample (48.3%, 73/151), patient declined to have the test done (35.8%, 54/151) and insurance cost concerns for patients (31.1%, 47/151), while 27.2% (41/151) reported no barriers. The survey demonstrated high awareness and compliance with MMR/MSI testing guidelines although universal testing rates seem to be suboptimal.

## 1. Introduction

Colorectal cancer (CRC) is the third most common type of cancer and the third leading cause of cancer-related deaths in the US [1]. Estimated cases of colon and rectal cancer in 2018 were 140,250, whereas estimated deaths attributed to colon and rectal cancers in 2018 were 50,630. The 5-year survival rates for CRC stage IIIb is about 64.6–76.9%, stage IIIc 45.5–61.8% and stage IV 7.4–14.2% [2]. CRC imposes a significant burden on the healthcare system with the direct costs of CRC accounting for close to 12% of all direct cancer costs, or about $14 million USD annually [3]. The current decline in incidence of and mortality from CRC [4] is principally a result of improvements in screening, rather than the result of major therapeutic advances [5].

In heritable CRC, there is a known family history of CRC and/or adenomatous polyps. In the case of hereditary CRC, however, there is a specific genetic mutation that has been identified. Lynch syndrome (previously called hereditary non-polyposis colorectal cancer) is the most common hereditary colon cancer syndrome [6]. It is essential to identify patients who potentially have Lynch syndrome, as not only they, but also family members, will benefit from screening and monitoring for CRC as well as for other extra-colonic tumors, particularly endometrial tumors [7].

MMR proteins are responsible for correcting strand alignment and base matching errors during DNA replication [6,8,9]. If one of these proteins is defective or non-functional, it is reflected in length alterations in microsatellites, or microsatellite instability (MSI) [6,10,11,12]. It is for this reason that cancers of this type are identified as being mismatch repair deficient (dMMR), and microsatellite instability high (MSI-H), as opposed to proficient MMR (pMMR) and MSI low (MSI-L) or microsatellite stable (MSS) [6,7,13]. Approximately 15% of all CRCs are associated with defects in the DNA mismatch repair (MMR) system [14]. Of these dMMR/MSI-H cancers, approximately 12–13% are sporadic CRCs, and 2–3% are inherited CRCs (Lynch syndrome).

New 2018 guidelines from the National Comprehensive Cancer Network (NCCN) on molecular testing in CRC recommend that MMR/MSI testing or analysis for deficient MMR protein expression be done in all patients with newly diagnosed CRC [15]. Very little is known about how any guidelines for molecular testing in CRC are translating into routine and real-life clinical practice. Furthermore, there have been no studies looking at physicians’ understanding of MMR/MSI testing specifically. However, according to prior research, it would appear that physicians tend to have inadequate discussions about family history of cancer, and therefore do not identify those patients who are appropriate candidates for genetic testing [16,17,18]. Other barriers to genetic testing identified by physicians have included lack of time and availability to make referrals, high financial costs of testing for patients, and absence of reimbursement through insurance [19].

New treatment options are now available for certain patients with dMMR/MSI-H tumors. Therefore, there is a need to assess MMR/MSI testing practices among physicians. Ultimately, this will assist in the understanding of how MMR/MSI testing can lead to optimally tailored treatment solutions for patients.

The objective of this study was to assess the knowledge and awareness of MMR/MSI testing and to understand MMR/MSI testing practices among US physicians.

## 2. Materials and Methods

This study consisted of two phases, a qualitative survey development phase and a quantitative survey deployment phase.

The first phase (qualitative phase) involved the development and pilot testing of the physician online surveys. The draft survey was developed based on a targeted literature review and exploratory interviews with physicians (5 oncologists, 5 surgeons and 5 pathologists) involved in CRC patient management. Interviews were conducted between December 2017 and March 2018. In total, 125 codes were used. More than 90% of these codes were identified within the first 10 interviews conducted, with only 9 new codes emerging during the final 5 interviews, indicating saturation.

The draft survey was then pilot tested and adjusted according to results of the pilot tests. During pilot testing, a new series of physicians (4 oncologists, 3 surgeons and 3 pathologists) completed the survey online, and were interviewed to assess their comprehension and acceptability of the survey. The final survey was subsequently generated.

The second phase (quantitative phase) involved sending the web link of the final survey to larger cohorts (*n* = 151 physicians) for collection of quantitative data about MMR/MSI testing. The questionnaire, developed in US English, primarily comprised close-ended items (Yes/No, Likert scale, etc.). The survey was designed for deployment via a web link.

Physicians were eligible to participate in the study if they were based in the US with over 5 years of experience (including fellowship training) on treating CRC patients, had a population of at least one mCRC patient in his/her routine in the past six months of practice in the US, had experience with the MMR/MSI test in CRC, and had ordered at least one MMR/MSI test in CRC in the past six months.

Physicians were invited to participate in all phases of the study through a market research recruitment agency which sourced members of their voluntary research panels and by networking. For phase two, members of oncology research panels were invited to take part in the survey. Physicians were compensated at fair market value rates for all phases of the study. All phases of this study were reviewed and approved by Quorum Review IRB.

### Statistical Analysis

Qualitative interview data were analyzed using thematic analysis with Atlas.ti. Concept saturation was documented to show that all the concepts that were important for the interviewees had been captured.

The quantitative online survey was analyzed descriptively. Results for all survey questions were descriptively summarized as the frequency and percentage of respondents selecting each answer. Descriptive statistics were calculated in the overall population, as well as by physician subgroups (physician specialty, type of practice, and years of practice). As there were no significant differences between type of practice and years of practice, these data are not shown.

Univariate and multivariate logistic regression models were fit to estimate (1) associations between physician characteristics and awareness of guidelines, and (2) associations between physician characteristics and routine ordering of MSI testing.

All statistical analyses were conducted using SAS version 9.4 or later for Windows.

## 3. Results

Physicians were recruited by pre-defined specialty quotas: oncologists (*n* = 91), pathologists (*n* = 45) and surgeons (*n* = 15). The online survey data was collected in July 2018. Physician characteristics are presented in Table 1.

Overall, awareness of MMR/MSI testing guidelines was high; 84.1% (127/151) of physicians were aware of existing guidelines on MMR/MSI testing for the management of patients with CRC (Table 2). Of those, 93.7% (119/127) followed published guidelines. The NCCN guidelines were followed by 67.2% (80/119) physicians. A substantially higher proportion of pathologists (31.4%, 11/35) than oncologists (5.5%, 4/73) and surgeons (9.1%, 1/5) followed the ASCP-CAP-AMP-ASCO guideline. Internal/institutional practice guidelines were followed by 20.5% (26/127) of physicians. There were no statistically significant relationships in physician characteristics and awareness of guidelines for either univariate or multivariate analyses.

Physicians most often decide to order the MMR/MSI test themselves (77.5%, 117/151), but there were differences between physician specialties (Table 2). Oncologists (89.0%) and pathologists (68.9%) most commonly decide to order the test themselves, whereas a multidisciplinary team most often orders the test for surgeons (66.7%). A total of 18.5% (28/151) have a physician from another specialty deciding to order the MMR/MSI test. A total of 68.9% (104/151) of physicians perform universal testing for all CRC patients. The MMR/MSI test is selectively ordered by 29.8% (45/151) of physicians.

**Figure 1:**
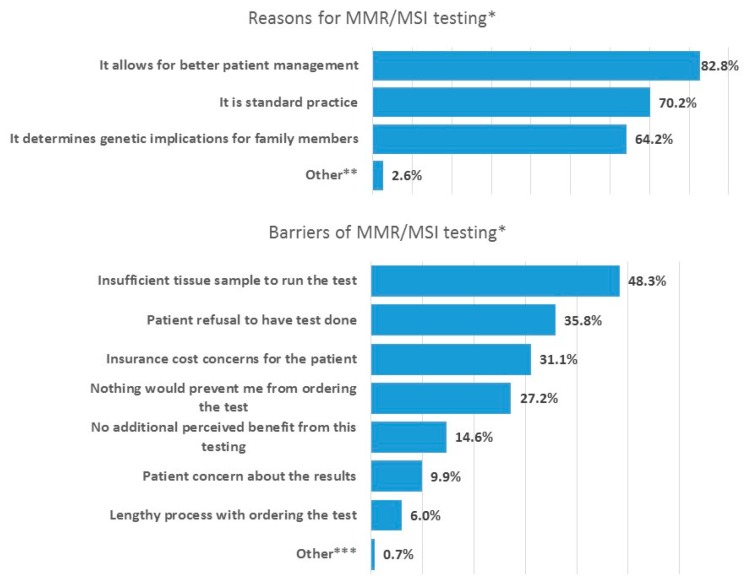
Reasons and barriers for ordering MMR/MSI testing—% of physicians agreeing with each statement. * Multiple selections were allowed. Thus, total percentage will not add up to 100%. ** Answer included: eligibility for certain therapies, and compliance with guidelines. *** Answer included: Patients do not go to third line.

The majority of physicians agreed that “it allows for better patient management” (82.8%, 125/151), “it is standard practice” (70.2%, 106/151), and “it determines genetic implications for family members” (64.2%, 97/151) were reasons to order the test (Figure 1). Physicians agreed with the barriers for ordering MMR/MSI testing were “insufficient tissue sample” (48.3%, 73/151), “patient refusal to have the test done” (35.8%, 54/151) and “insurance cost concerns for the patients” (31.1%, 47/151). Pathologists were more concerned with the barrier of “insufficient tissue sample to run the test” than oncologists and surgeons. Only 6.0% (9/151) considered “lengthy process with ordering the test” to be a barrier. 27.2% (41/151) indicated “nothing would prevent me from ordering the test”.

Interpretation of the test result is most often done individually (67.5%, 102/151) or as part of a multidisciplinary team (31.1%, 47/151), while 12.6% (19/151) indicated that another physician interprets (Table 3). Oncologists and pathologists most often interpret the test results individually. Surgeons most often interpret the test results as part of a multidisciplinary team. Whether treatment recommendations based on the test results were made individually, as part of a multidisciplinary team or by another physician varied across the different specialties. Oncologists indicated they make treatment recommendations individually (86.8%, 63/91) or as part of multidisciplinary team (37.4%, 34/91). Pathologists make treatment recommendations either as part of a multidisciplinary team (37.8%, 17/45) or let another physician make recommendations (48.9%, 22/45). All surgeons indicated they sometimes make treatment recommendations as part of multidisciplinary team (100%, 15/15) and some also make sometimes treatment recommendations individually (26.7%, 4/15).

The approach to discussing the MMR/MSI test with patients varied across physician specialties (Table 3). Oncologists discuss MMR/MSI testing and potential impact on patient care both before and after the test (57.1%, 52/91) or only after the test (37.4%, 34/91). Surgeons most commonly discuss both before and after the test (60.0%, 9/15) or only after the test (33.3%, 5/15). Pathologists most often let another specialty physician communicate with patients (84.4%, 38/45).

**Figure 2:**
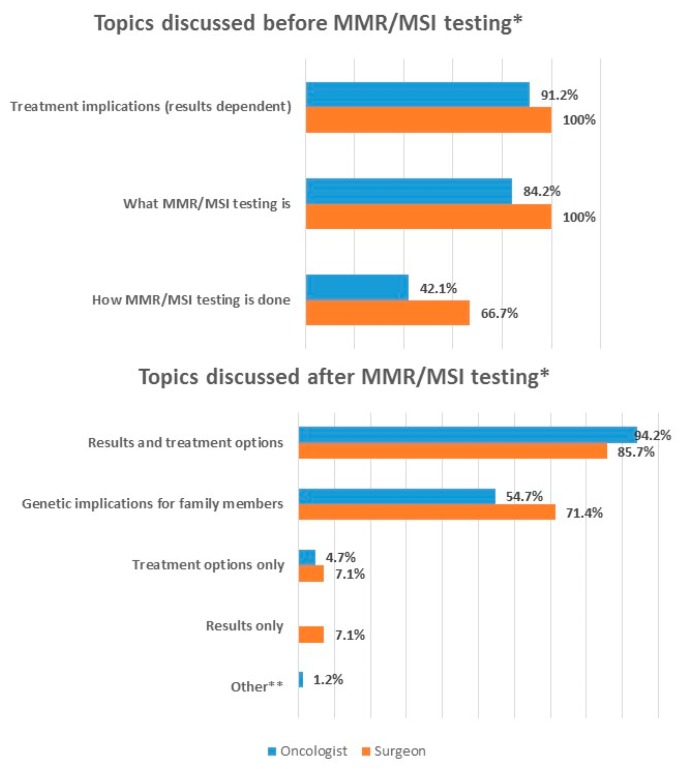
Physician discussion with patients—% of physicians agreeing with each statement. * Multiple selections were allowed. Thus, total percentage will not add up to 100%. ** Referral to genetic counselling.

It is primarily oncologists and surgeons who discuss various issues with patients before the testing is done. Before the testing is done, oncologists discuss what MMR/MSI testing is (84.2%, 48/57) and treatment implications (91.2%, 52/57) (Figure 2. How MMR/MSI testing is done is also discussed but to a lesser extent (42.1%, 24/57). Similarly, surgeons discuss what MMR/MSI testing is (100%, 9/9) and treatment implications (100%, 9/9), and how MMR/MSI testing is done (66.7%, 6/9).

After the test is done, it is again oncologists and surgeons who discuss with patients. Oncologists discuss both results and treatment options (94.2%, 81/86), and genetic implications for family members (54.7%, 47/86) (Figure 2). Few oncologists discuss treatment options only (4.7%, 4/86). Surgeons discuss both results and treatment options (85.7%, 12/14), and genetic implications for family members (71.4%, 10/14).

## 4. Discussion

This survey of 151 US physicians with different specialties, all involved in the management of patients with CRC, showed that their level of awareness of MMR/MSI testing guidelines in CRC patients was high. The NCCN guidelines are being followed by most physicians. Universal testing for all CRC patients was performed by 68.9% (104/151) physicians, while 29.8% (45/151) selectively orders the test for some CRC patients. Based on the recent 2018 NCCN guidelines endorsing universal testing of all newly diagnosed CRC patients, the results of this study suggest that there is significant room for improvement [15]. The survey results further indicate that there is agreement among physicians that MMR/MSI testing allows for better patient management, and the vast majority agreed MMR/MSI testing is part of the standard clinical tests available in their practice. Overall, there was a high perception of agreement to the benefits of MMR/MSI testing in patient management. We did not find any differences between physician specialty, practice type and years of practice for awareness of MMR/MSI testing guidelines.

Although we showed a high level of awareness in our survey, in a recent database analysis, using data from the National Cancer Database, Shaikh et al. showed poor MMR testing rates (ranging between 28.2% and 43.1%) in CRC patients, and non-adherence to testing guidelines in young adults [20]. This may somehow be linked to our 29.8% (45/151) of physicians who reported to selectively order the test for some CRC patients. Other survey studies conducted a few years ago show a similar pattern. In a Canadian survey study, 21.5% of respondents were unaware of whether they had access to MMR immunohistochemistry [21]. A US survey study reported 71% of NCI comprehensive cancer centers, 36% of American Colleague of Surgeons-accredited community hospital comprehensive cancer centers, and only 15% of community hospital centers perform routine tumor MSI testing for patients with Lynch syndrome [14]. Parikh et al. surveyed US physicians for genetic screening for Lynch syndrome in newly diagnosed stage II CRC patients and found there is undertesting related to Lynch screening and overtesting involving molecular tests [22].

Only 27.2% of physicians in our survey reported that no barriers existed to prevent them from ordering testing, indicating that a large proportion of the physicians raised some concerns. Physicians indicated that insufficient tissue sample to perform the test was a key barrier. In addition, patient refusal to have the test done and insurance cost concerns were also considered barriers. These barriers relating to the patient perspective emphasize the need to better inform and educate patients, and also to find ways to improve cost coverage. The cost of MMR/MSI testing varies from a few hundred dollars to a few thousand dollars, and also depends on the number of genes tested [23]. The cost of the test is covered by many US health insurance companies and coverage by Medicare and Medicaid varies between different states [24]. However, as the coverage and reimbursement are subject to the benefit plan, American Society of Clinical Oncology (ASCO) recommends that the physician verify the cost coverage of the testing components with the patient’s insurance plan prior to ordering the test. Despite a general coverage of the cost of the test across the US, our survey indicates that there is still concerns about costs and that there is a need for improvement. Notably, few physicians reported the process of ordering the test to be a barrier. Similarly, in a multinational survey, Ciardiello 2016 surveyed 859 physicians across South America, Europe and Western Asia and their use of biomarker testing, with 43% of the sample treating CRC patients [19]. The use of biomarker testing was high in this sample, with cost or non-reimbursement reported as the most common reasons for not using biomarker testing.

There were differences between physician specialties in terms of the discussion of test results with patients, where pathologists most often have another physician discuss with the patient, which is expected due to the nature of their profession. Oncologists and surgeons discuss with patients both before and after the test, which includes discussion of test results and treatment options. This is important given the increased importance of recognizing patient preferences in treatment decisions [25].

Limitations include a small sample size, in particular for the surgeon specialty, and the inherent limitations of self-reported data. The sample may not be representative of the entire US physician population, although efforts were made to recruit across different specialties, types and years of practice. The results should therefore be interpreted with caution in terms of being representative across the entire spectrum of clinical practice. Furthermore, we did not collect information on actual rates of ordering the MMR/MSI test.

Future research should investigate MMR/MSI testing practices in other cancers, including endometrial, renal cell, and more, and also in other country settings. Educational tools to address barriers for MMR/MSI testing could be explored. Awareness of MSI status, i.e., germline versus somatic, is important to investigate.

In conclusion, this survey demonstrated high awareness and compliance with MMR/MSI testing guidelines although universal testing rates seem to be suboptimal. Addressing the key physician barriers to testing along with increased communication and education on the benefits of testing may help to enhance testing rates.

## Figures and Tables

**Table 1:** Physician characteristics.

	*n* = 151; *n* (%)
Specialty	
Oncologist	91 (60.3%)
Pathologist	45 (29.8%)
Surgeon/Surgical Oncologist	15 (9.9%)
Practice *	
Hospital	65 (43.0%)
Private practice (independent)	76 (50.3%)
Private practice (academic)	20 (13.2%)
Other **	2 (1.3%)
Years Practicing	
Greater than/equal to 15 years	96 (63.6%)
Less than 15 years	55 (36.4%)

* Multiple selections were allowed. Thus, total percentage will not add up to 100%. ** Other practice types specified included: Integrated Delivery Network (*n* = 1) and Academic University Comprehensive Cancer Center (*n* = 1).

**Table 2:** MMR/MSI testing guideline awareness and management.

	All Physicians (*n* = 151); *n* (%)	Physician Specialty		
		Oncologist (*n* = 91); *n* (%)	Pathologist (*n* = 45); *n* (%)	Surgeon/Surgical Oncologist (*n* = 15); *n* (%)
Are you aware of specific MMR/MSI testing guidelines?				
Yes	127 (84.1%)	74 (81.3%)	42 (93.3%)	11 (73.3%)
If yes, which guidelines? *				
Published guidelines	119 (93.7%)	73 (98.6%)	35 (83.3%)	11 (100%)
NCCN	80 (67.2%)	49 (67.1%)	26 (74.3%)	5 (45.5%)
ASCP-CAP-AMP-ASCO	16 (13.4%)	4 (5.5%)	11 (31.4%)	1 (9.1%)
ESMO	2 (1.7%)	1 (1.4%)	1 (2.9%)	-
Other **	29 (24.4%)	22 (30.1%)	2 (5.7%)	5 (45.5%)
Internal/Institutional practice guidelines	26 (20.5%)	11 (14.9%)	12 (28.6%)	3 (27.3%)
Who decides to order MMR/MSI testing? *				
Myself	117 (77.5%)	81 (89.0%)	31 (68.9%)	5 (33.3%)
A physician from another specialty	28 (18.5%)	7 (7.7%)	19 (42.2%)	2 (13.3%)
Multidisciplinary team	51 (33.8%)	20 (22.0%)	21 (46.7%)	10 (66.7%)
Other ***	9 (6.0%)	6 (6.6%)	1 (2.2%)	2 (13.3%)
Are you routinely ordering MMR/MSI testing for all CRC patients?				
Yes, I perform universal testing for all my CRC patients	104 (68.9%)	61 (67.0%)	31 (68.9%)	12 (80.0%)
No, I selectively order MMR/MSI testing for some of my CRC patients	45 (29.8%)	30 (33.0%)	12 (26.7%)	3 (20.0%)
Other ****	2 (1.3%)	-	2 (4.4%)	-

* Multiple selections were allowed. Thus, total percentage will not add up to 100%. ** Answers included: no specific guideline mentioned, testing for all patients, and other non-relevant comments. *** Answers included: pathologist, reflex testing. **** Answers included: based on published criteria, at request of another physician.

**Table 3:** Interpretation and discussion of MMR/MSI test results.

	All Physicians (*n* = 151); *n* (%)	Physician Specialty		
		Oncologist (*n* = 91); *n* (%)	Pathologist (*n* = 45); *n* (%)	Surgeon/Surgical Oncologist (*n* = 15); *n* (%)
Do you interpret the lab clinical test results of the MMR/MSI testing? *				
Yes, individually	102 (67.5%)	63 (69.2%)	35 (77.8%)	4 (26.7%)
Yes, as part of a multidisciplinary team	47 (31.1%)	30 (33.0%)	7 (15.6%)	10 (66.7%)
No, another physician interprets	19 (12.6%)	10 (11.0%)	6 (13.3%)	3 (20.0%)
Do you make treatment recommendations based on the lab results of the MMR/MSI testing? *				
Yes, individually	92 (60.9%)	79 (86.8%)	9 (20.0%)	4 (26.7%)
Yes, as part of a multidisciplinary team	66 (43.7%)	34 (37.4%)	17 (37.8%)	15 (100%)
No, another physician makes recommendations	22 (14.6%)	-	22 (48.9%)	-
Do you discuss with patients what MMR/MSI testing is and how it can impact patient care?				
Yes, discuss with patient before the test only	6 (4.0%)	5 (5.5%)	1 (2.2%)	-
Yes, discuss with patient after the test only	42 (27.8%)	34 (37.4%)	3 (6.7%)	5 (33.3%)
Yes, discuss with patient before and after the test	63 (41.7%)	52 (57.1%)	2 (4.4%)	9 (60.0%)
No, a physician from another specialty communicates	39 (25.8%)	-	38 (84.4%)	1 (6.7%)
Other **	1 (0.7%)	-	1 (2.2%)	-

* Multiple selections were allowed. Thus, toal percentage will not add up to 100%. ** Answer included: oncologist treating the patient discuss.
